# Thromboelastometry Identified Alteration of Clot Stabilization and Factor XIII Supplementation Need in a Patient with Decompensated Liver Disease Undergoing Liver Biopsy

**DOI:** 10.1155/2018/6360543

**Published:** 2018-08-29

**Authors:** Tomaz Crochemore, Felicio Aragão Savioli

**Affiliations:** Hospital Leforte, Department of Critical Care, São Paulo, SP, Brazil

## Abstract

Liver disease has been considered the prototype of hemorrhagic disease. Disorder in any component of coagulation system can lead to hemorrhage. Deficiency of factor XIII may impair clot strength and clot stabilization and can be accessed by thromboelastometry. We report a case of a patient with a rapid evolution of liver disease who underwent a liver biopsy. Thromboelastometry was performed, evidencing impairment of clot stability. This clotting disorder was corrected with factor XIII concentrate after unsuccessful administration of antifibrinolytic drugs and hepatic biopsy was performed without hemorrhagic complications.* Case Presentation*. We report the case of a previously healthy 38-year-old man, who presented to our emergency department with clinical signs of rapid progression of acute liver failure. The laboratory tests revealed platelets of 142x10^3^/mm3, plasma fibrinogen concentration of 221 mg/dl, increased international nationalized ratio (INR 1.9), total bilirubin of 3.9mg/dl, direct bilirubin of 2.3mg/dl, ALT 751U/l, and AST 540U/l without acute bleeding. A liver biopsy was indicated. Based on the results of the thromboelastometry, Tranexamic Acid was administered to correct hyperfibrinolysis followed by factor XIII concentrate to correct factor XIII deficiency. Thromboelastometry was normal despite conventional coagulation tests were still altered. So, liver biopsy was performed with no signs of bleeding and without need of further transfusion.* Conclusion*. Thromboelastometry may be considered a useful, feasible, and safe tool to monitor and manage coagulopathy in patients with liver disease, with the potential advantage of helping avoid unnecessary transfusion in such patients.

## 1. Introduction

Coagulopathy in liver disease has been considered as one of the major challenges in the area of thrombosis and hemostasis [1]. In recent years liver disease has been proven to be no longer an exclusively hemorrhagic disease; otherwise, clinical signs of thrombosis can be found [2]. The clinical manifestation depends on imbalance between pro- and anticoagulant drivers as well as pro- and antifibrinolytic components [1].

Traditionally, patients with decompensated liver disease who exhibit abnormal results of conventional coagulation tests (CCT) have been subjected to prophylactic transfusion of blood components before undergoing an invasive procedure. However, CCT are poor predictors of bleeding, and allogeneic blood components transfusion is associated with poor outcome.

Studies in patients with liver disease have suggested that thromboelastometry (ROTEM®) may be a better test in predicting risk of bleeding than coagulation conventional tests and platelet count [3]. Thromboelastometry accesses the entire process of clot formation including the initiation, maximum firmness, and stabilization of the clot [4]. Specific coagulation disorder as dysfibrinogenemia, hyperfibrinolysis, and factor XIII deficiency can be identified early [5].

Therefore, thromboelastometry used for identifying coagulopathy and guided hemostatic therapy could be considered in cases of patients with liver disease with risk of bleeding and need to undergo an invasive procedure such as liver biopsy. Consequently, our objective was to describe a case of a patient with fast onset liver disease with unknown cause whose hepatic biopsy procedure was successfully guided by thromboelastometry. Coagulopathy was corrected with coagulation factor concentrate and hemostatic drugs without transfusion of allogeneic blood component.

## 2. Case Presentation

**Figure 1:**
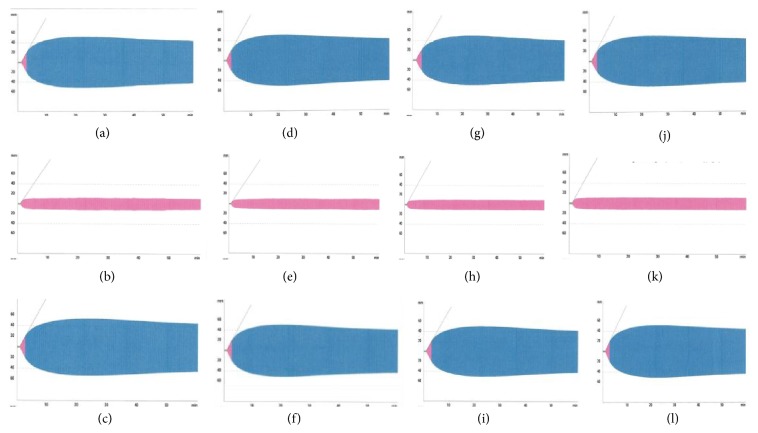
Sequential thromboelastometry (ROTEM). ((a), (b), and (c)) Thromboelastometry analysis at the hospital admission ((a) EXTEM, (b) FIBTEM, and (c) APTEM). ((d), (e), and (f)) Second thromboelastometry analysis performed after Tranexamic Acid administration ((d) EXTEM, (e) FIBTEM, and (f) APTEM). ((g), (h), and (i)) Third thromboelastometry analysis performed after additional administration of Tranexamic Acid ((g) EXTEM, (h) FIBTEM, and (i) APTEM). ((j), (k), and (l)) Thromboelastometry analysis performed after factor XIII concentrate (Fibrogammin® P, CSL Behring, Marburg, Germany) ((j) EXTEM, (k) FIBTEM, and (l) APTEM).

We report the case of a previously healthy 38-year-old man, Afro-Brazilian, with no previous medical records. He presented to our emergency department with an acute onset of abdominal pain, jaundice, fever, nausea, weakness, and malaise. His arterial blood pressure was 90/50mmHg, heart rate was 90 bpm, axillary temperature was 35°C, and he was dehydrated. The laboratory examinations revealed serum creatinine of 0.8 mg/dl, platelets of 142 x 10^3^/mm3, serum fibrinogen of 221 mg/dl, increased international nationalized ratio (INR 1.9), total bilirubin of 3.9mg/dl, direct bilirubin of 2.3mg/dl, ALT 751U/l, AST 540U/l, ceruloplasmin of 17 mg/dl, ferritin of 3200 ng/dl, iron of 276mcg/dl, TIBC (total iron-binding capacity) of 267mcg/dl, transferrin saturation of 103%, hemoglobin (Hb) of 14 g/dl, and hematocrit (Ht) of 41,3% without acute bleeding. US (ultrasound) showed signs of inflammation and liver fibrosis as well as iron overload. A liver biopsy was indicated. First ROTEM test showed in EXTEM CT 80s, CFT 105s, alfa-angle 70°, MCF 52 mm, and ML 37%; FIBTEM MCF 13 mm and APTEM MCF 53 mm and ML 20% (Table 1/Figures 1(a), 1(b), and 1(c)). Tranexamic Acid 1 g was administered to correct hyperfibrinolysis. Second ROTEM test presented improvement in the hyperfibrinolysis but not completely, so another 1 g of Tranexamic Acid was administered with EXTEM CT 64s, CFT 105s, alfa-angle 69°, MCF 51 mm, and ML 24%; FIBTEM MCF 11 mm, APTEM MCF 51 mm, and ML 20% (Table 1/Figures 1(d), 1(e), and 1(f)). The third ROTEM test showed persistence of maximum lysis above 15% with EXTEM CT 83s, CFT 133s, alfa-angle 66°, MCF 50 mm, and ML 21%; FIBTEM MCF 10 mm and APTEM MCF 51 mm and ML 19% (Table 1/Figures 1(g), 1(h), and 1(i)). This time 1500U of factor XIII concentrate was administrated to correct factor XIII deficiency and fourth ROTEM test was normal with EXTEM CT 64s, CFT 122s, alfa-angle 68°, MCF 50 mm, and ML 15%; FIBTEM MCF 12 mm and APTEM MCF 51 mm and ML 15% (Table 1/Figures 1(j), 1(k), and 1(l)). Thromboelastometry was normal despite CCT were still altered. So, liver biopsy was performed which succeeded with no signs of bleeding and without need of further transfusion. He was discharged from the hospital 5 days after admission.

## 3. Discussion

Liver disease is often associated with coagulation disorders. CCT are usually quite altered and therefore transfusion of blood components has been a frequent practice. Transfusion of allogeneic blood products is associated with a higher risk of infection, pulmonary failure, and mortality [6, 7]. However, CCT present late results and are punctual since they access only 5% of thrombin generation [8]. Thus, CCT are limited to predict bleeding or guide transfusion therapy with blood components.

In 2001, the concept of the cell-based coagulation model described by Monroe and Hoffman pointed out the role of the surface of cell membrane for the generation of thrombin. This complex process of clot formation is triggered from the release of the tissue factor by the endothelium. In this cellular model, the clot formation process goes through 3 stages: thrombin generation including initiation and amplification phases; maximum clot firmness or propagation phase; and last phase the clot stabilization [9]. Both the fibrinolytic system and factor XIII are considered the determinants of clot stabilization.

Rotational thromboelastometry is a laboratory method performed at the bedside that accesses the entire process of clot formation. First ROTEM identifies the initial process involving the coagulation factors of both the extrinsic and intrinsic pathway to generate thrombin. Subsequent phase consists in a fibrin clot formation from fibrinogen, platelets, and also factor XIII when clot reaches maximum firmness [4]. The final phase of stabilization involves the fibrinolytic system as well as factor XIII activated responsible for cross-linking of fibrin [10].

Discovered in 1940s, blood coagulation factor XIII (F XIII) is a transglutaminase that is present in plasma, activated by the combined action of thrombin and Ca2+. Its function includes cross-linking of fibrin monomers between themselves to generate a stable fibrin strand, but also cross-linking of fibrinogen with *α*2 antiplasmin, a potent plasmin inhibitor, to protect the fibrin clot from fibrinolysis [11, 12]. Thus, factor XIII acts on the clot firmness but mainly on clot stabilization. FXIII deficiency is not detected by routine coagulation assays and needs to be searched by measuring FXIII levels with quantitative assay [4, 5]. Inherited FXIII deficiency with undetectable FXIII activity is associated with a severe bleeding tendency. FXIII levels below 50% are associated with a smaller *α*2AP incorporation and enhanced clot lysis [10, 13].

Thromboelastometry allows the evaluation of factor XIII deficiency in two different ways. First, factor XIII deficiency can be thought of if the maximum clot firmness (MCF) in FIBTEM remains reduced even after adequate replacement of fibrinogen. The other possibility is when the maximum lysis found in EXTEM is above 15%; however, the maximum lysis remains above 15% to APTEM, even after the administration of antifibrinolytic drugs [14]. In both situations, the quantitative measurement of factor XIII is not mandatory, since thromboelastometry accesses the quality of formation and stabilization of the clot. Sudden quantitative reduction in the measurement of factor XIII may compromise the stabilization of the clot, without critically low levels of factor XIII being detected necessarily.

In this reported case, traditionally the patient with acute liver disease and INR 1.9 would probably be transfused with fresh frozen plasma because of the high risk of bleeding during liver biopsy. However, thromboelastometry performed identified impairment in clot stabilization. According to the first ROTEM test, Tranexamic Acid 1 g was administered to correct hyperfibrinolysis defined by ML EXTEM > 15% (Figures 1(a), 1(b), and 1(c)). There was partial improvement in the second test that still showed hyperfibrinolysis parameters 8 h after the first test (Figures 1(d), 1(e), and 1(f)). An additional dose of 1 g Tranexamic Acid was administered, but without changing the ML in both EXTEM and APTEM. At this time, the third ROTEM test showed ML EXTEM above 15%; then 1500U of factor XIII concentrated was administered (Figures 1(g), 1(h), and 1(i)). The control ROTEM was normal and liver biopsy was undergone without any bleeding complication, confirming the usefulness of factor XIII infusion to increase clot stability confirmed by thromboelastometry in a situation where acquired factor XIII deficiency is expected (Figures 1(j), 1(k), and 1(l)).

## 4. Conclusion

This case reports the use of thromboelastometry for diagnosis of specific coagulation disorder in patients with liver disease with high risk of bleeding and need to undergo an invasive procedure such as liver biopsy.

Thromboelastometry identified factor XIII deficiency and guided the therapy with factor XIII concentrate correcting clotting disorder. Factor XIII replacement improved the clot stabilization and liver biopsy was performed without bleeding complications. No transfusion of allogeneic blood component was needed. Nevertheless, additional studies are needed to define the ability of thromboelastometry to identify factor XIII deficiency in liver disease patients, but also to define the role of factor XIII concentrate to improve clot stabilization.

## Figures and Tables

**Table 1:** Sequential analysis of thromboelastometry (ROTEM®) parameters.

PARAMETERS	1^ST^ ROTEM	2^ND^ ROTEM	3^RD^ ROTEM	4^TH^ ROTEM
EXTEM CT (S)	80	64	83	64
EXTEM CFT (s)	105	105	133	122
EXTEM *α*-ANGLE(°)	70	69	66	68
EXTEM MCF (mm)	52	51	50	50
EXTEM ML (%)	37	24	21	15
FIBTEM MCF (mm)	13	11	10	12
APTEM MCF (mm)	53	51	51	51
APTEM ML (%)	20	20	19	15

## References

[1] Tripodi A., Primignani M., Chantarangkul V. (2009). An imbalance of pro- vs anti-coagulation factors in plasma from patients with cirrhosis. *Gastroenterology*.

[2] Tripodi A. (2017). Hemostasis in Acute and Chronic Liver Disease. *Seminars in Liver Disease*.

[3] Crochemore T., De Toledo Piza F. M., Silva E., Corrêa T. D. (2015). Thromboelastometry-guided hemostatic therapy: An efficacious approach to manage bleeding risk in acute fatty liver of pregnancy: A case report. *Journal of Medical Case Reports*.

[4] Crochemore T., Piza F. M., Rodrigues R. d., Guerra J. C., Ferraz L. J., Corrêa T. D. (2017). A new era of thromboelastometry. *Einstein (São Paulo)*.

[5] Abuelkasem E., Lu S., Tanaka K., Planinsic R., Sakai T. (2016). Comparison between thrombelastography and thromboelastometry in hyperfibrinolysis detection during adult liver transplantation. *British Journal of Anaesthesia*.

[6] Sarani B., Dunkman W. J., Dean L., Sonnad S., Rohrbach J. I., Gracias V. H. (2008). Transfusion of fresh frozen plasma in critically ill surgical patients is associated with an increased risk of infection. *Critical Care Medicine*.

[7] Silliman C. C., McLaughlin N. J. D. (2006). Transfusion-related acute lung injury. *Blood Reviews*.

[8] Mann K. G. (2003). Thrombin Formation. *Chest*.

[9] Hoffman M., Monroe D. M. (2001). A cell-based model of hemostasis. *Thrombosis and Haemostasis*.

[10] Rijken D. C., Uitte De Willige S. (2017). Inhibition of Fibrinolysis by Coagulation Factor XIII. *BioMed Research International*.

[11] Martinuzzo M., Barrera L., Altuna D. (2016). Effects of Factor XIII Deficiency on Thromboelastography. Thromboelastography with Calcium and Streptokinase Addition is more Sensitive than Solubility Tests. *Mediterranean Journal of Hematology and Infectious Diseases*.

[12] Fraser S. R., Booth N. A., Mutch N. J. (2011). The antifibrinolytic function of factor XIII is exclusively expressed through *α*2-antiplasmin cross-linking. *Blood*.

[13] Kattula S., Byrnes J. R., Martin S. M. (2018). Factor XIII in plasma, but not in platelets, mediates red blood cell retention in clots and venous thrombus size in mice. *Blood Advances*.

[14] Theusinger O. M., Baulig W., Asmis L. M., Seifert B., Spahn D. R. (2010). In vitro factor XIII supplementation increases clot firmness in rotation thromboelastometry (ROTEM®). *Thrombosis and Haemostasis*.

